# Activating One/Two‐Photon Excited Red Fluorescence on Carbon Dots: Emerging n→*π* Photon Transition Induced by Amino Protonation

**DOI:** 10.1002/advs.202207566

**Published:** 2023-02-05

**Authors:** Qing Zhang, Fengqing Wang, Ruoyu Wang, Junlan Liu, Yupengxue Ma, Xiaoru Qin, Xiaoxia Zhong

**Affiliations:** ^1^ State Key Laboratory of Advanced Optical Communication Systems and Networks Key Laboratory for Laser Plasmas (Ministry of Education) School of Physics and Astronomy Shanghai Jiao Tong University Shanghai 200240 P. R. China; ^2^ Department of Food Science and Technology School of Agriculture and Biology Shanghai Jiao Tong University Shanghai 200240 P. R. China; ^3^ Institute of Molecular Medicine (IMM) Renji Hospital Shanghai Jiao Tong University School of Medicine Shanghai Jiao Tong University Shanghai 200240 P. R. China

**Keywords:** carbon quantum dots, molecular state, photoluminescence mechanism, reaction sediments

## Abstract

Due to the complicated nature of carbon dots (CDs), fluorescence mechanism of red fluorescent CDs is still unrevealed and features highly controversial. Reliable and effective strategies for manipulating the red fluorescence of CDs are urgently needed. Herein, CDs with one‐photon excited (622 nm, QYs ≈ 17%) and two‐photon (629 nm) excited red fluorescence are prepared by acidifying *o*‐phenylenediamine‐based reaction sediments. Systematic analysis reveals that the protonation of amino groups increases the particle surface potential, disperse the bulk sediments into nano‐scale CDs. In the meanwhile, amino protonation of pyridinic nitrogen (–N=) structure inserts numerous n orbital energy levels between the *π* → π* transition, narrows the gap distance for photon transition, and induces red fluorescence emission on CDs. Present research reveals an effective pathway to activate CDs reaction sediments and trigger red emission, thus may open a new avenue for developing CDs with ideal optical properties and promising application prospects.

## Graphical abstract

Due to the advantages of long wavelength emission, near‐infrared (NIR) fluorescent Carbon Dots (CDs) attract broad interest. However, strategies for manipulating NIR fluorescence of CDs are urgently needed. Here, the authors reveal an effective pathway to activate one‐photon and two‐photon NIR emission on CDs, providing a new avenue for developing CDs with ideal optical properties.

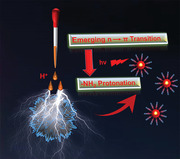

## 1 Introduction

Carbon dots (CDs), as a new emerging member of the carbon nano family, features divers advantages of small particle size (10 nm), fascinating tunable photoluminescence from ultraviolet to near‐infrared, excellent chemical and biology stability, and low biological toxicity. These merits make the CDs as a supernova in broad frontier research and practical applications like selective sensing, bio‐imaging, biomedicines, and photoelectronics.^[^ ^1^ ^]^

Among the abundant species of CDs, the Red‐CDs featuring long wavelength fluorescence emission raised tremendous research attention due to the outstanding properties of low water absorption, high penetration depth in aqueous solution and biological tissues, and low photon‐induced bio‐damages.^[^ ^2^ ^]^ However, the fluorescence luminescence mechanism of CDs is still unrevealed. The complicated theoretical hypothesis and difficulty realized experimental evidence make it still hard to manipulate the fluorescence features of CDs (Table S1–S3, Supporting Information).^[^ ^2^, ^3^ ^]^As such, traditional Red‐CDs preparation usually involves the usage of un‐predicted carbonaceous precursors, harsh preparation conditions, and complicated purification processes.^[^ ^1^, ^4^ ^]^ These exceptional requirements make the Red‐CDs preparation often encountered with confusing precursor selection, strong device‐dependency, and time‐consuming purification process.

Furthermore, as a byproduct, reaction sediments produced during the traditional CDs preparation processes has occupied the majority mass (can up to 90%) of the reaction products.^[^ ^5^ ^]^ Unfortunately, the sediments produced are regarded as useless carbon bulks and being discarded directly without further utilization in most of the researches.^[^ ^6^ ^]^ Thus, as for now, the structure formation and functional composition relation between sediments and CDs are usually ignored without further investigation. This not only results in wasting the CDs reaction products and low CDs productivity, but also causes the potential danger of environmental pollution. Moreover, this may further make us lose the opportunity to explore another new world of CDs, in which the fascinating CDs with novel functionalities may aggregate and presented in a unique formation.

**Figure 1:**
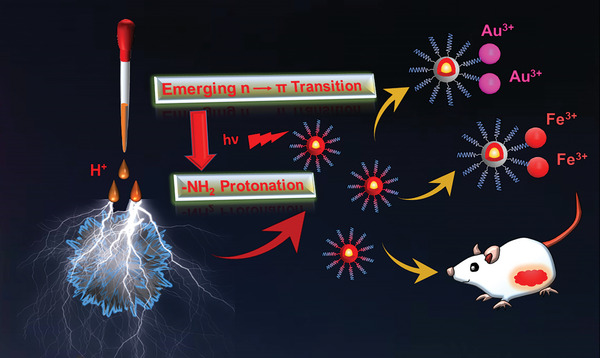
Schematic illustration of fluorescence mechanism for activating one‐photon and two‐photon excited red fluorescence on Carbon Dots via the emerging new n→*π* photon transition induced by protonated amino groups, and the applications in selective metal‐ions sensing and in vivo bio‐imaging.

Herein an CDs with one/two‐photon excited red fluorescence emission are facilely prepared by acidifying the reaction sediment produced by the preparation process of conventional *o*‐phenylenediamine CDs (See **Figure** **1**). Systematic analysis reveals that the protonation of amino functional groups remarkably changes the molecular state of fluorophore, causes prominent variations in both surface charge distribution and internal energy band structure. In detail, the protonated amino functional groups increase the particle surface Zeta potential, raises the repulsion force between particle, disperses the bulk sediments into nano‐scale CDs. In the meanwhile, the protonated amino groups remarkably change the internal energy band structure of CDs by inserting numerous n orbital energy levels between the *π* → π* transition gap. This effectively narrows the gap distance for photon transition, and eventually induces one‐photon excited red emission at 622 nm (Full width at half maximum, FWHM, 33 nm) and two‐photon excited red emission at 629 nm. Further research find that the prepared Red‐CDs features excellent metal‐ions detection ability in extremely acidic condition, which can selective recognize the Au^3+^ and Fe^3+^ ions among 11 kinds of different metal ion solution including Zn^2+^, Na^+^, K^+^, Fe^2+^, Fe^3+^, Cu^2+^, Ag^+^, Cd^2+^, Au^3+^, Mg^2+^, Ni^2+^ at pH 0. Furthermore, the Red‐CDs also exhibit promising prospects in in vivo bio‐imaging. Collectively, this research gives an insight for re‐activating sediments produced by CDs preparation and identify the essential role of functional group protonation on the red FL emission, which may open a new pathway for preparing Red‐CDs with novel application.

## 2 Results

### 2.1 Morphology, Structure and Composition

**Figure 2:**
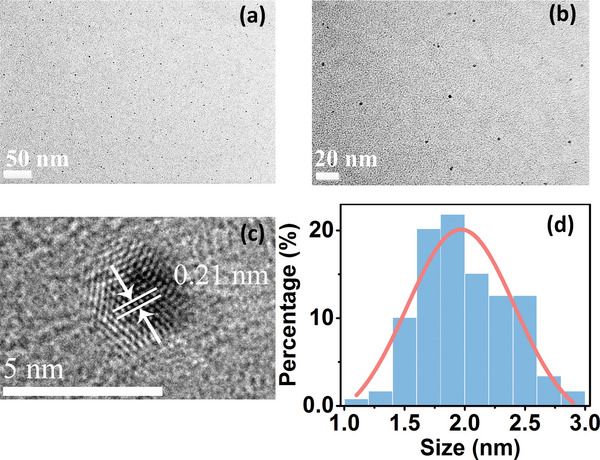
Transmission electron microscopy (TEM) images of Red‐CDs at a) 50 nm, b) 20 nm, and c) 5 nm resolution. d) Size distribution of Red‐CDs.

The morphology of prepared Red‐CDs is characterized by transmission electron microscopy (TEM) image with different resolution at 50, 20, and 5 nm as shown in **Figure** **2**a–c. Figure 2a,b reveals that the prepared Red‐CDs are well dispersed in water. High‐resolution TEM images of Red‐CDs revealed in Figure 2c indicate that the prepared Red‐CDs have a lattice distance of 0.21 nm attributing to the (1,0,0) plane of graphite.^[^ ^1a^ ^]^ Size distribution of Red‐CDs is analyzed according to the obtained TEM images and the results are shown in Figure 2d. It can be observed that the average particle size of Red‐CDs is around 1.9 nm and particle size distribution of Red‐CDs ranges from 1.0 to 3.0 nm. The structure formation of Red‐CDs is characterized using Raman spectra analysis and the results is shown in Figure S1 of the Supporting Information. The prepared Red‐CDs primary features three peaks locating at 1252, 1379, and 1546 cm^−1^ which respectively attributed to the vibration of disordered *sp^3^* structure (D,1252 cm^−1^, 1379 cm^−1^) and in‐plane graphitic *sp^2^* structure (G, 1546 cm^−1^).^[^ ^7^ ^]^ The ratio of *I*_D_/*I*_G_ is 0.83 between 1379 and 1546 cm^−1^. The prominent vibration intensity of *sp^2^* structure revealed by *I*_D_/*I*_G_ illustrate the graphitic nature of Red‐CDs.

**Figure 3:**
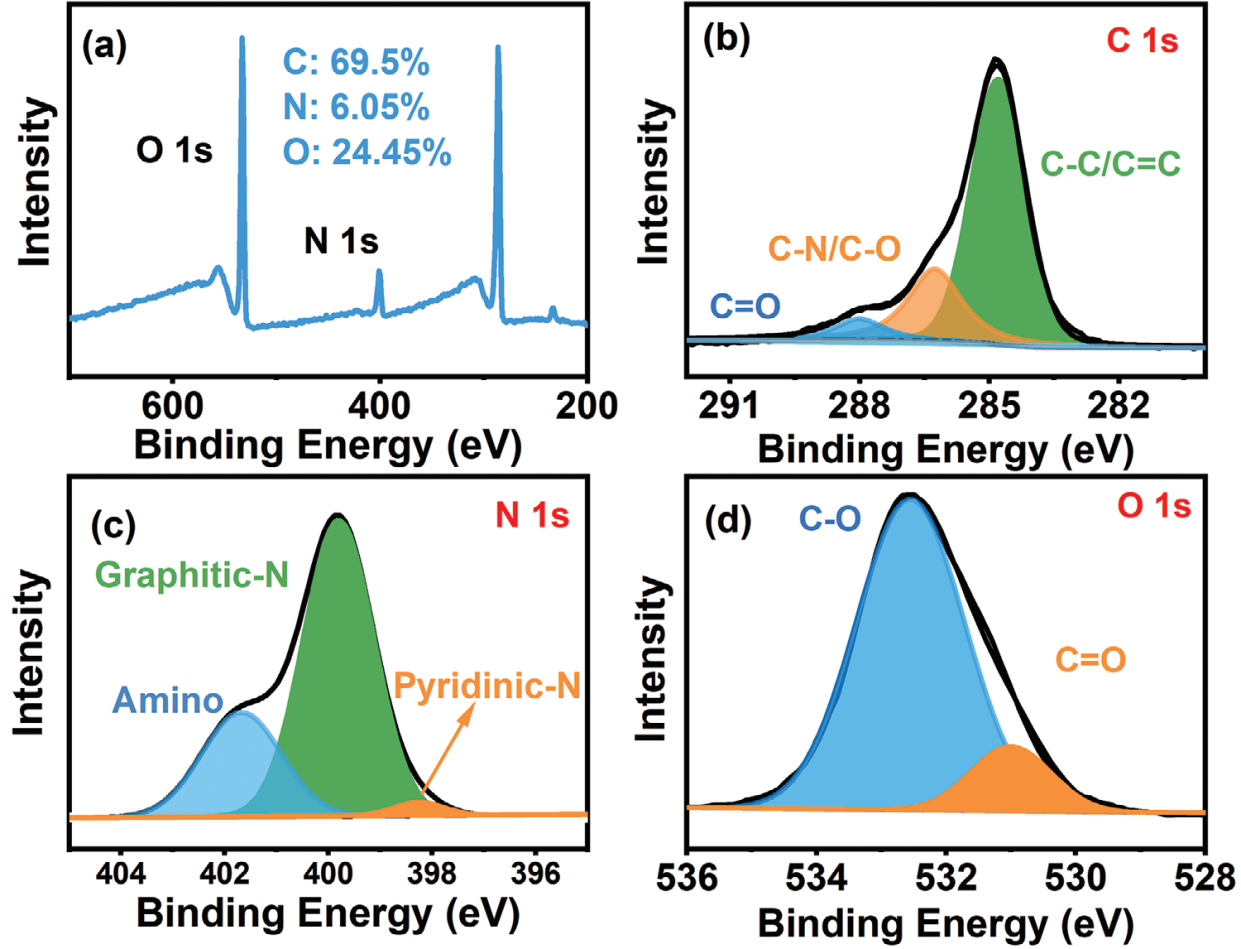
X‐ray photoelectron spectroscopy (XPS) of Red‐CDs. a) Full‐scale XPS spectra, b) high‐resolution C 1s XPS spectra, c) high‐resolution N 1s XPS spectra, d) high‐resolution O 1s XPS spectra of Red‐CDs.

The composition of prepared Red‐CDs is characterized by X‐ray photoelectron spectroscopy (XPS) analysis. As shown in **Figure** **3**a, the Red‐CDs are mainly composed by 3 kinds of element including carbon (69.5%), nitrogen (6.05%), and oxygen (24.45%). High‐resolution XPS spectra of C 1s (See Figure 3b) revealed that 3 kinds of carbon valence shown as C=C (284.7 eV), C‐N/C‐O (286.3 eV), and C=O (288.0 eV) are distributing on the Red‐CDs. High‐resolution XPS spectra of N 1s (See Figure 3c) illustrate that the nitrogen valence states shown as pyridinic nitrogen (398.2 eV), graphitic nitrogen (399.8 eV), amino (401.6 eV) are contained by Red‐CDs. High‐resolution XPS spectra of O 1s are revealed in Figure 3d. In accordance with the results of C 1s XPS spectra, the oxygen valence of C=O (530.9 eV), C–O (532.5 eV) are resolved in Red‐CDs.^[^ ^1^, ^3^ ^]^

### 2.2 Optical Properties

**Figure 4:**
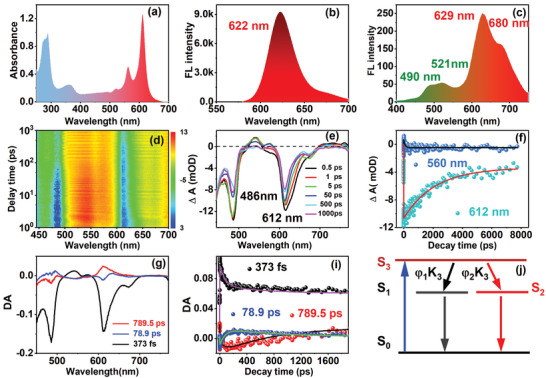
Optical properties of prepared Red‐CDs. a) UV‐Vis spectra, b) one‐photon fluorescence spectra of CDs excited at 612 nm, c) Two‐photon fluorescence spectra of Red‐CDs excited by 808 nm femtosecond laser, d) 3D transient absorption (TA) spectra of Red‐CDs, e) TA spectra of Red‐CDs at decay time ranges from 0.5 ps to 1000 ps, f) Kinetic traces of TA spectra at probe wavelength of 560 and 612 nm, g) Decay associated difference spectra obtained by singular value decomposition (SVD), h) Global fitting results of TA principal component decay kinetic traces, i) Photoluminescence transition model proposed for Red‐CDs.

UV‐Vis spectra of Red‐CDs are recorded by dissolving the CDs products in deionized water. As shown in **Figure** **4**a, Red‐CDs feature five absorption bands respectively located ≈280, 365, 520, 560, and 612 nm. Absorption bands around 280 and 365 nm are attributed to the *π* → π* transition of the aromatic C=C bonds within Red‐CDs. Absorption bands ≈520, 560, and 612 nm are assigned to the n → π* transition of the heterocycle ring, C=O, or C‐N groups.^[^ ^8^ ^]^ Fluorescence emission spectra of Red‐CDs are revealed in Figure 4b, and Figures S2, S3 and Table S4 of the Supporting Information. The Red‐CDs exhibits red fluorescence (FL) emission centered at 622 nm (Quantum Yields, QYs 17%) with an excitation‐independent behavior. The full width at half maximum (FWHM) of the fluorescence spectra is 33 nm, which is much narrower than that of most presented reports. Up‐conversion FL spectra of Red‐CDs under 808 nm fs laser are detected and shown in Figure 4c. As is revealed, the Red‐CDs feature four up‐conversion emission peak respectively locating around 490, 521, 629, and 680 nm. As shown in Figure S4, to investigate the up‐conversion processes, FL spectra of Red‐CDs under varied excitation intensity are recorded and the relationship between FL intensity and excitation power also is analyzed. Figure S5 of the Supporting Information revealed that the up‐conversion FL intensity of Red‐CDs increased in well linear relationship (*R* = 0.98) with the femtosecond laser power. Thus, this indicates that a two‐photon excitation process has occurred during the up‐conversion processes. As such, UV‐Vis and fluorescence spectra characterization illustrate that the prepared Red‐CDs features both red absorbance at 612 nm, one‐photon and two‐photon excited red FL emission around 629 nm.

To investigate the PL mechanism of Red‐CDs, energy level structure, transition dynamics of the excited state on Red‐CDs are characterized and analyzed using transient absorption (TA) spectra.^[^ ^9^ ^]^ The 3D TA spectra of Red‐CDs with probe wavelength ranging from 440 to 700 nm in decay time between 0.5 ps and 7.7 ns are detected under excitation of 400 nm and demonstrated in Figure 4d. Negative TA band between 470 and 493 nm (blue area), positive TA spectra ranging from 503 to 590 nm and negative TA band between 600 and 700 nm are detected and observed. TA spectra at varied decay time are presented in Figure 4e. In accordance with the results of 3D TA spectra, the decay spectra clearly exhibit the absorption peaks locating at 486, 541, 576, 612, and 672 nm. According to the UV‐Vis absorption spectra and steady fluorescence spectra, negative peak of 486, 622, and 672 nm belongs to stimulated emission (SE) (See Figure S6, Supporting Information).^[^ ^10^ ^]^ Positive peaks of 541 and 576 nm are caused by the excited state absorption （ESA）. The negative peak at 612 nm is ascribed to the ground state bleaching (GSB). The absorption peak at 612 nm gradually decrease with the increment of decay time from 0.5 to 1000 ps. Due to the short lifetime of stimulated emission, the negative peak at 486 nm has no obvious attenuation compared with the negative peak at 612 nm within the initial 5 ps, but the attenuate is faster after then (See Figure S7, Supporting Information).^[^ ^10^ ^]^ Kinetic traces of Red‐CDs at 486, 560, and 612 nm are revealed and shown in Figure S8 of the Supporting Information and Figure 4f.

To reveal the relaxation processes of the excited state, a singular value decomposition (SVD) was carried out based on the TA spectra data. Three principal components are revealed with significance coefficients of 1.29, 0.19, and 0.10. As demonstrated in Figure 4g, three decay‐associated difference spectra (DADS) are obtained. Further Global fitting analysis reveals the DADS features with fitted lifetime of 373 ± 300 fs, 78.9 ± 74 ps, and 789.5 ± 670 ps (See Figure 4h). According to the physical processes of photoluminescence, a transitions model (See Figure 4i) is supposed and used to illustrate relaxation mechanism of the excited state. There are three excited states for Red‐CDs: excited state (S_3_), metastable excited state (*S*_1_), and metastable excited state (S_2_).^[^ ^3^, ^11^ ^]^ When excited with external light, hot carriers in the excited state (*S*_3_) emerge on Red‐CDs. Then, these hot carriers undergo a relaxing process through scattering of electron, optical phonon, and acoustical phonon within 373 ± 300 fs. After the relaxation process, the hot carriers reach to the metastable excited state (*S*_1_) with the probability 83%, and metastable excited state (*S*_2_) with probability of 17%. Hot carriers in the metastable excited state (*S*_1_) transit back to the ground state (*S*_0_) within 78.9 ± 74 ps through a non‐radiative process e.g. collisions. Hot carriers in the metastable excited state (*S*_2_) transit back to the ground state (S_0_) within 789.5 ± 670 ps in a radiative way via recombination of electrons‐holes.^[^ ^3d^ ^]^ The TA spectra reveal the prepared Red‐CDs features simple energy‐level structure and single red PL center leading to narrowed red emission.^[^ ^2d^ ^]^

### 2.3 Red Photoluminescence Mechanism

**Figure 5:**
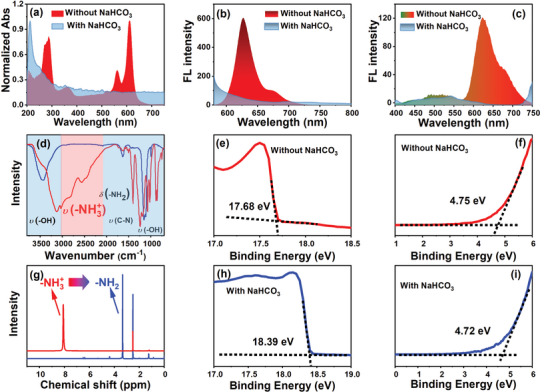
Photoluminescence mechanism of Red‐CDs. a) UV‐Vis, b) One‐photon FL spectra excited under 560 nm; c) two‐photon FL spectra excited by 808 nm fs laser, d) FTIR spectra; e), f) UPS spectra of Red‐CDs treated without NaHCO_3_. g) H^1^‐NMR spectra of Red‐CDs treated with (blue line) or without NaHCO_3_ (red line); h), i) UPS spectra of Red‐CDs treated with NaHCO_3_.

As reminded by the results of TA spectra analysis, a systematic comparison between Red‐CDs and deprotonated Red‐CDs are carried out. The deprotonated Red‐CDs are prepared by treating the Red‐CDs with saturated sodium bicarbonate (NaHCO_3_) aqueous solution. As shown in **Figure** **5**a, UV‐Vis spectra analysis reveal that, after deprotonation, the multitude absorption peaks of Red‐CDs at 280, 365, 520, 560, and 612 nm all disappear. Only the absorption peak locating around 215 nm is retained.

UV‐Vis absorption edges are also calculated shown in Figures S9 and S10 of the Supporting Information. As shown in Figure S9, Red‐CDs features absorption edges located at 258 313. 408, 589, and 638 nm. Figure S10 reveals that only the absorption ≈246 nm was retained. According to the relationship between absorption edge and transition energy gap shown as *E*_g_ = 1240/*λ*_edge_, the transition band gap width of Red‐CDs at 4.8, 3.99, 3.04, 2.10, and 1.94 eV disappear (See Figure S9, Supporting Information). A new absorption transition gap emerges on the deprotonated Red‐CDs at 5.0 eV (See Figure S10, Supporting Information). As the deprotonation treatment directly affects the molecular state of fluorophore on CDs. Thus, the variation of absorption features caused by the deprotonation indicate that both *π* → π* and n → π* transition channels of Red‐CDs are strongly affected by the molecular state of fluorophore. Especially, the n energy orbital and n → π* transition on Red‐CDs even vanished after deprotonation.

FL spectra demonstrated in Figure 5b reveal that, the red one‐photon FL emission of Red‐CDs at 622 and 675 nm both vanish after deprotonation treatment of NaHCO_3_. Similar results are observed in two‐photon FL spectra (See Figure 5c), red two‐photon FL emission of Red‐CDs at 629 and 680 nm disappeared. Weak visible FL emission of Red‐CDs located at 490 and 521 nm are retained. According to the formula of E = h*ν*, it can be calculated that the photon transition with bands gaps of 1.99 eV (622 nm),1.97 eV (629 nm),1.83 eV (675 nm) and 1.82 eV (680 nm) vanishes and only the band gaps of 2.53 (490 nm) eV and 2.38 eV (521 nm) are retained after deprotonation treatment. The strong influence of deprotonation on red emission and its related energy band gaps illustrate that the molecular state of organic fluorophore plays an essential role in the FL emission of Red‐CDs.

To identify the fluorophore species responding for red FL emission, Fourier transform infrared spectroscopy (FTIR), nuclear magnetic resonance hydrogen spectrum (H‐NMR), and ultraviolet photoelectron spectroscopy (UPS) analysis are further performed. As shown in Figure 5d, the FTIR spectra of Red‐CDs (red line) feature strong vibration at 3471 cm^−1^ attributing to the stretching of hydroxyl (–OH). Dispersive vibration bands range from 2000 to 3200 cm^−1^ involving peaks located at 3128, 3029, 2797, and 2559 cm^−1^ are caused by the stretching vibration of group NH_3_^+^.^[^ ^12^ ^]^ Vibration peaks at 1634 cm^−1^ are ascribed to asymmetric deformation vibration of NH_3_^+^. The 3 weak vibration peaks respectively located at 1572, 1532, and 1501 cm^−1^ are caused by the vibration of aromatic rings.^[^ ^13^ ^]^ Vibration peaks located at 1402 cm^−1^ are attributed to the symmetric deformation vibration of NH_3_^+^. The absorption peak at 1233 cm^−1^ is due to the stretching vibration of C–N structure. Absorption peaks at 1189 and 1020 cm^−1^ are respectively ascribing to the asymmetric and symmetric stretching vibration of SO_3_ within HSO_4_^−1^.^[^ ^12^ ^]^ The absorption peak located at 878 cm^−1^ is due to the stretching vibration of S‐OH. FTIR spectra of Red‐CDs with deprotonation treatment of NaHCO_3_ are revealed as blue line in Figure 5d. Stretching vibration of hydroxyl (–OH) is located at 3462 cm^−1^. In comparison with the case of Red‐CDs, the serials stretching vibration peaks of NH_3_^+^ ranging between 2000 and 3200 cm^−1^ disappear. Asymmetric deformation vibration of NH_3_^+^, asymmetric stretching and symmetric stretching vibration of SO_3_ belongs to Red‐CDs all disappear together. Asymmetric deformation vibration of –NH_2_ groups locates at 1635 cm^−1^. Stretching vibration of C–N structure is retained and shifted into 1158 cm^−1^. As such, the results of FTIR analysis reveal that analogs of protonated aromatic amine are prominently distributing on Red‐CDs. The protonation treatment of NaHCO_3_ has largely removed the proton (H^+^) on the NH_3_^+^ groups of Red‐CDs.

Nuclear magnetic resonance hydrogen spectra (H‐NMR) of Red‐CDs are detected and illustrated in Figure 5g and Figures S11 and S12 of the Supporting Information. The H‐NMR spectra of Red‐CDs (red line in Figure 5g) feature dominate peak at 8.07 ppm corresponding to the vibration of NH_3_^+^ groups. Zoomed picture shown in Figure S11 of the Supporting Information further reveals peaks at 7.2, 6.9, and 6.7 ppm, attributing vibration of aromatic rings. Zoomed picture shown in Figure S12 of the Supporting Information further confirms the vibration of hydrogen ion within pyridinic *N*‐doped aromatic structures in accordance with the results of N 1s XPS spectra. H‐NMR spectra of Red‐CDs with deprotonation treatment are shown in the blue line of Figure 5g and Figure S13 of the Supporting Information. In comparison with that of Red‐CDs, vibration peaks of NH_3_^+^ groups vanish. Vibration peaks of aromatic rings are retained and shifted to 6.5 and 6.33 ppm. New vibration peak of ‐NH_2_ emerges at 4.39 and 3.33 ppm. In the meanwhile, Zeta potential analysis reveals that the produced Red‐CDs features a potential of 38.5 mV (See Figure S14, Supporting Information). When encountered with NaHCO_3_ deprotonation, the zeta potential of deprotonated Red‐CDs reduces into −3.6 mV (See Figures S15 and S16, Supporting Information). In accordance with the analysis results of FTIR analysis. The H‐NMR and Zeta potential results confirm the distribution of protonated aromatic amine groups on Red‐CDs and further evidences the transition of NH_3_^+^ → NH_2_ caused by the NaHCO_3_ deprotonation treatment.

To further understand the protonation effect on the band structure of Red‐CDs, ultraviolet photoelectron spectroscopy (UPS) analysis is carried out under excitation of He 1 light source with incident energy (*E*_incident photon_) of 21.2 eV. Full‐scale UPS spectra of Red‐CDs are revealed and shown in Figure S17 of the Supporting Information. High‐resolution UPS spectra are shown in Figure 5e and Figure 5f. The secondary electron cutoff (*E*_cutoff_) and near Fermi energy edge (*E*_Fermi_) are revealed at 17.68 and 4.75 eV, respectively. According to the calculation equation: *E*_HOMO_ = *E*_incident –_ (*E*_coutoff –_ *E*_Fermi_), the energy level of the highest occupied molecular orbital (HOMO) is 8.27 eV.^[^ ^14^ ^]^ Full‐scale UPS spectra of Red‐CDs with NaHCO_3_ deprotonation are shown in Figure S18 of the Supporting Information. High‐resolution UPS spectra of deprotonated Red‐CDs are revealed in Figure 5h and Figure 5i. *E*_coutoff_ and *E*_Fermi_ energy of deprotonated Red‐CDs are 18.39 and 4.72 eV. Under similar calculation, the HOMO energy of deprotonated Red‐CDs is calculated as 7.53 eV. Thus, based on the results of UPS analysis, the deprotonation treatment has leaded to the decrement of HOMO energy level on Red‐CDs.

**Figure 6:**
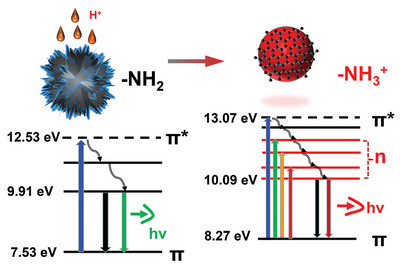
Proposed photoluminescence mechanism of the prepared Red‐CDs. Activated aromatic amino (‐NH_2_) groups on Red‐CDs introduce numerous n energy orbital levels, narrows the band gaps of excited state energy transition, then triggering the red fluorescence emission.

Collective with the results of UV‐vis absorption, FL emission, FTIR, NMR, Zeta potential, and UPS analysis, it can be concluded that the positively charged ‐NH_3_^+^ groups on CDs introduce numerous n energy orbital levels, narrows the band gaps of the photon energy transition, then triggering the red fluorescence emission (See **Figure** **6**).

### 2.4 Applications

Owing to the excellent long wavelength fluorescence of the prepared Red‐CDs, the CDs are further applied in multitude of applications of selective metal ions detection, and red bio‐imaging.

**Figure 7:**
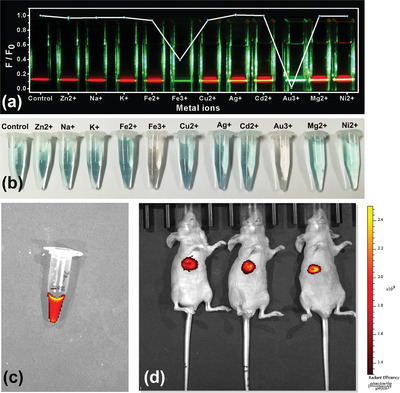
Applications of prepared Red‐CDs. a) Selective detection of Au^3+^ and Fe^3+^ ions under excitation of 532 nm. b) photograph of the ions treated with Red‐CDs. c) Light emitting photograph of Red‐CDs under excitation of 610 nm; d) In vivo bio‐imaging of nude mice.

As a proof of metal ions detection, the produced Red‐CDs of 200 µg mL^−1^ are treated with 11 kinds of metal ions including Zn^2+^, Na^+^, K^+^, Fe^2+^, Fe^3+^, Cu^2+^ Ag^+^, Cd^2+^, Au^3+^, Mg^2+^, Ni^2+^ at a concentration of 2 mM. The variation of FL intensity of Red‐CDs is shown in **Figure** **7**a. It is interesting to be observed that the FL intensity of Red‐CDs in Fe^3+^ and Au^3+^ ions solution separately reduces 60% and 99%, indicating the produced Red‐CDs feature high selectivity toward these two kinds of ions at pH 0. The photograph shown in Figure 7b reveals that the absorption color of the metal‐ions Red‐CDs solution changes from blue to brownness under visible light. The color changes of metal‐ions solution mixed with Red‐CDs were also confirmed by UV‐Vis absorption analysis. As revealed by Figure S19 of the Supporting Information, the treatment of Fe^3+^ and Au^3+^ ions directly decreased the absorption of Red‐CDs at 560 and 612 nm, and then increases the visible light absorption ranging from 400 to 500 nm. According to the previous reports, this phenomenon may be caused by the fast electron transfer between the strong electron‐donating ‐NH_2_ group of Red‐CDs and electron‐deficient Fe^3+^ and Au^3+^ ions, which changes the molecular state of protonation, resulting in directly nonluminous electron‐hole recombination and lead to the decrement of fluorescence intensity.^[^ ^15^ ^]^ In accordance with the results of pure environment, FL intensity of Red‐CDs in Fe^3+^ and Au^3+^ ions solution with inorganic salts interference of ZnSO_4,_ Na_2_SO_4_, KCl, MgSO_4_, NiSO_4_, and CuSO_4_ also separately reduces 60% and 99% (See Figure S20, Supporting Information). Little fluorescence decrement is observed between the inorganic salt interference group of inorganic salts mixture and control group of Red‐CDs. As most of the pollutant detection need to be analyzed under the strong acid condition to ensure the ions state of metals and few labels can work normally under these harsh environments, thus, the above‐mentioned quenching results indicate our Red‐CDs can also work as a promising fluorescent label in detecting Au^3+^ and Fe^3+^ ions.

In vivo bio‐imaging is performed by subcutaneously injecting the Red‐CDs dissolved in phosphate buffer solution (PBS) at a concentration of 200 µg mL^−1^. Before injection, the nude mouse is first kept fasting for 24 h to avoid the background fluorescence caused by the fluorescent food residue. After injection of 30 min, the bio‐imaging is performed under excitation of 610 nm using PerkinElmer in vivo imaging system. As shown in Figure 7c,d, the Red‐CDs samples and nude mice injected with Red‐CDs exhibit bright fluorescence in the under excitation. The result shows that the signal‐to‐noise of the CDs in bio‐imaging is 3.6. This indicates the prepared Red‐CDs are promising in the application of bio‐imaging.

These novel properties illustrate that the produced CDs feature promising prospect in the practical application of heavy metal‐ions detection and bio‐medical imaging.

## 3 Discussion and Conclusion

Due to the unrevealed photoluminescence (PL) mechanism, it is still now hard to controllable activating red fluorescence on Carbon Dots. In this research, Red‐CDs with narrowed one‐photon excited red emission and two‐photon excited red emission are facilely prepared by acidifying the reaction sediment of conventional o‐phenylenediamine based CDs. Combined analysis of FL, TA, UV‐Vis, FTIR, and NMR reveal that protonation of amino groups remarkably change the internal energy band structure of CDs by inserting numerous n orbital energy levels between the *π*→π* transition gap and activate the red fluorescence emission on Carbon Dots. As revealed by the results of XPS and NMR, the prepared Red‐CDs indeed features abundant structure of pyridinic nitrogen (–N=) on the surface, thus it may be concluded that the protonation of amino conjugated with pyridinic nitrogen (–N=) may trigger the red FL emission on CDs. According to the density functional theory calculation performed by Chiang et al, as the results of amino protonation (–NH_2_ → ‐NH_3_^+^), bandgap of photon transition decreases and fluorescence emission red shift in the case of pyridinic nitrogen (–N=) doped Carbon Dots.^[^ ^16^ ^]^ In the case of graphitic and pyrrolic nitrogen‐doped Carbon Dots, the bandgap of photon transition remains unchanged or increased, and the fluorescence emission remains unchanged or blue‐shifted. Thus, it can be confirmed that the protonation of amino group conjugated with pyridinic nitrogen (–N=) structure narrows the band gap of photon transition and activates red FL emission on CDs. Additional applications demonstrate that the prepared Red‐CDs feature promising prospects in both heavy‐metal ions detection and in vivo bio‐imaging. The findings present an effective pathway to regulate the long wavelength red fluorescence emission on CDs and also indicate a new way for recycling the byproducts of traditional CDs preparation. Therefore, it may open a new avenue for developing CDs with ideal optical properties and promising application prospects.

## 4 Experiment Section

### Preparation of Red‐CDs

A 280 mg o‐phenylenediamine (AR, Shanghai, China) were dissolved into 20 mL deionized water, and added into 50 mL Teflon reactor. Then, the Teflon reactor with o‐phenylenediamine solution was heated at 220 °C for 6 h. After the thermal treatment, the liquid supernatant was discarded and the Teflon lining with sediment was also washed with deionized water for three times to remove the soluble particles. The sediments on the medial wall were dissolved in the 1.5 mL H_2_SO_4_ solution and then the dissolved in 8 mL deionized water. After protonation treatment for 30 min, the obtained Red‐CDs H_2_SO_4_ mixture was further centrifuged at 12000 g for 30 min. The sediments were washed with deionized water for three times by centrifuging at 12000 g for 30 min. Then the sediments are dissolved in 50 mL of deionized water and filtrated with an 0.22 µm filter. The solution of Red‐CDs was further freezing‐dried for 3 days and stored at 4 °C.

### Apparatus

Morphology and composition of the prepared Red‐CDs were characterized by transmission electron microscope (TEM), (JEOL, JEM‐2100F, Japan), and X‐ray photoelectron spectroscopy (XPS), (AXIS ULTRA DLD, Kratos, Japan) analysis and Raman spectra analysis (Renishaw, UK). Optical properties of the prepared Red‐CDs were characterized by steady UV‐Vis light absorption (Avaspec‐2048‐2‐USB2, Avantes, Netherland), steady fluorescence emission spectra (F‐2700 Hitachi, Japan), and femtosecond transient absorption spectrum (Ultrafast, Helios, USA). The Structure of Surface functional groups of Red‐CDs are identified using FTIR, (Nicolet 6700, Thermo Scientific, USA), and hydrogen nuclear magnetic resonance (700 MHz, Avance III, Germany). In vivo bio‐images were performed using in vivo imaging system (PerkinElmer, USA).

### Measurement of Quantum Yields (QYs)

The quantum yields of the prepared Red‐CDs were measured by referring to the QYs of rhodamine B dissolved in ethanol (85%). In detail, the prepared Red‐CDs are dissolved in deionized water with an absorbance below 0.1. The rhodamine B dissolved in ethanol also with absorbance below 0.1. Then, both UV‐Vis absorption and FL emission spectra are recorded in the same detecting condition. The QYs were calculated according to the following equation^[^ ^17^ ^]^:

(1) $$\Phi_{\mathrm{sm}}=\Phi_{\mathrm{st}}\left(\frac{\mathrm{Gra}d_{\mathrm{sm}}}{\mathrm{Gra}d_{\mathrm{st}}}\right)\left(\frac{\eta_{\mathrm{sm}}^{2}}{\eta_{\mathrm{st}}^{2}}\right)$$

in which the “Φ_sm_” indicates the QYs of Red‐CDs, “Φ_st_” indicates the QYs of rhodamine B. Grad_sm_ represents the integration of Red‐CDs FL intensity vs UV‐Vis absorption. Grad_st_ represents the integration of rhodamine B FL intensity vs UV‐Vis absorption. ‘*η*_sm_’ indicates the refractive index of Red‐CDs solution. ‘*η*_st_’ indicate the refractive index of rhodamine B solution.

### Selective Metal Ions Detection at Extremely Acid Condition

The mental‐ions selectivity of Red‐CDs was investigated by exploring the FL evolution within 11 common metal ion solutions at a concentration of 2 mM. Stock solution (0.04M, pH 0, adjusted by H_2_SO_4_) of metal ions were prepared from corresponding salts of ZnSO_4_, Na_2_SO_4_, FeSO_4_, Fe (NO_3_)_3_, CdSO_4_, HAuCl_4,_ AgNO_3_, KCl, MgSO_4_, NiSO_4_ and CuSO_4_. Red‐CDs at final concentration of 200 µg·mL^−1^ were also dissolved in the pH 0 H_2_SO_4_ solution as test solutions. A 150 µL stock solution of metal ions mixed with 3 mL test solutions for 24 hours and then, the FL spectra were measured and compared with that of control groups.

### In Vivo Bio‐Imaging

Nude mice (BKL: BALB/c‐nu/nu) were purchased from the experimental animal center in Shanghai. After food fasting for 24 h, the bio‐imaging was performed by subcutaneously injecting 150 µL of Red‐CDs dissolved in PBS at a concentration of 200 µg·mL^−1^. After injection of 30 min, the bio‐imaging is carried out under excitation of 610 nm using PerkinElmer in vivo imaging system. The animal experiments were reviewed and approved by Animal Care and Use Committee of Shanghai Jiao Tong University (SYXK‐2018‐0028).

## Conflict of Interest

The authors declare no conflict of interest.

## Author Contributions

X.X.Z and Q.Z conceived the idea and supervised the project. Q.Z designed the experiment and conducted most of the experiments involved in CDs preparation and characterization. F.Q.W and L.J.L conducted the experiments of in vivo fluorescent imaging and helped with the manuscript revision. R.Y.W performed the two‐photon fluorescence spectra detection and analysis. Y. P. X. M and X.R.Q performed the experiments of metal‐ions detection.

## Supporting information

Supporting Information

## Data Availability

The data that support the findings of this study are available from the corresponding author upon reasonable request.
