# Supplementary material for: Activating One/Two‐Photon Excited Red Fluorescence on Carbon Dots: Emerging n→π Photon Transition Induced by Amino Protonation
Source: Adv Sci (Weinh). 2023 Feb 5;10(11):2207566. doi: 10.1002/advs.202207566 (PMC10104635; doi:10.1002/advs.202207566)
Supplement: Supplementary file 1 — Supporting Information [file ADVS-10-2207566-s001.pdf]

ADVANCED SCIENCE
Open Access

Supporting Information

for *Adv. Sci.*, DOI 10.1002/advs.202207566

Activating One/Two-Photon Excited Red Fluorescence on Carbon Dots: Emerging n→π Photon Transition Induced by Amino Protonation

Qing Zhang, Fengqing Wang, Ruoyu Wang, Junlan Liu, Yupengxue Ma, Xiaoru Qin and Xiaoxia Zhong**

Supplementary Materials for

Activating Red Two-photon Fluorescence on Carbon Dots: Emerging n→π Photon Transition Induced by Amino Protonation

Qing Zhang, Fengqing Wang, Ruoyu Wang, Junlan Liu, Yupengxue Ma, Xiaoru Qin, Xiaoxia Zhong**

**Corresponding author. Email: xxzhong@sjtu.edu.cn;qingzhang@sjtu.edu.cn.*

Supplementary Figure and Table

Figure caption:
Fig. S 1 | Raman spectra of the prepared Red-CDs.

Fig. S 2 | Fluorescence spectra of prepared Red-CDs excited under different wavelength varied from 480 nm to 610 nm.

Fig. S 3 | (a) Fluorescence QYs standard curve of Rhodamine B (R^2=0.99); (b) Fluorescence QYs curve of Prepared Red-CDs (R^2=0.99).
Fig. S 4 | Upconversion fluorescence spectra of the prepared Red-CDs.
Fig. S 5 | Relationship between upconversion fluorescence intensity and laser power (R=0.98).
Fig. S 6 | Proposed energy-level transition diagram.
Fig. S 7 | kinetic traces of transient absorption spectra at probe wavelength of 486 nm (curve fitted with standard deviation of 0.00178).
Fig. S 8 | Steady fluorescence spectra of Red-CDs under excitation of 400 nm.
Fig. S 9 | Absorption edge of prepared Red-CDs.

Fig. S 10 | Absorption edge of prepared Red-CDs treated with $NaHCO_3$ solution.
Fig. S 11 | Zoomed H^1-NMR spectrum of Red-CDs.
Fig. S12 | Zeta potential data of the Red-CDs treated without $NaHCO_3$
Fig. S 13 | Zeta potential of the Red-CDs treated with $NaHCO_3$
Fig. S 14 | Zeta potential data of the Red-CDs treated with $NaHCO_3$
Fig. S 15 | Zoomed H^1-NMR spectrum of Red-CDs between 6.85 ppm to 7.8 ppm.
Fig. S 16 | Zoomed H^1-NMR spectrum of prepared Red-CDs treated with $NaHCO_3$ solution.
Fig. S 17 | Full scale UPS spectra of prepared Red-CDs.
Fig. S 18 | Full scale UPS spectra of prepared Red-CDs treated with $NaHCO_3$ solution.
Fig. S 19 | Variations of UV-Vis absorption during the selective detection of Au^{3+} and Fe^{3+}.
Fig. S 20 | Selective (a) Au^{3+} ions and (b) Fe^{3+} ions detection under inorganic salts interference of $ZnSO_4$, Na_2SO_4, KCl, $MgSO_4$, $NiSO_4$ and $CuSO_4$.

Table caption:
Table S1. Comparison of the FWHM between Red-CDs and the latest literature.
Table S2. Comparison of one-photon Red emission of Carbon Dots between previous research and our work.
Table S3 Comparison of two-photon Red emission mechanism of CDs between previous research and our work.
Table S4. Fluorescence QYs data of Rhodamine B and Red-CDs.

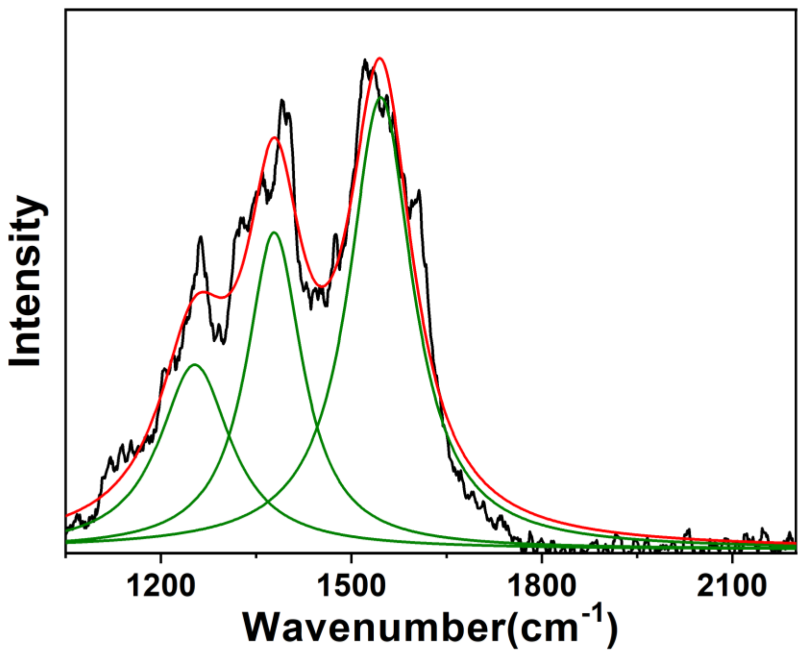

Fig. S 1 | Raman spectra of the prepared Red-CDs.

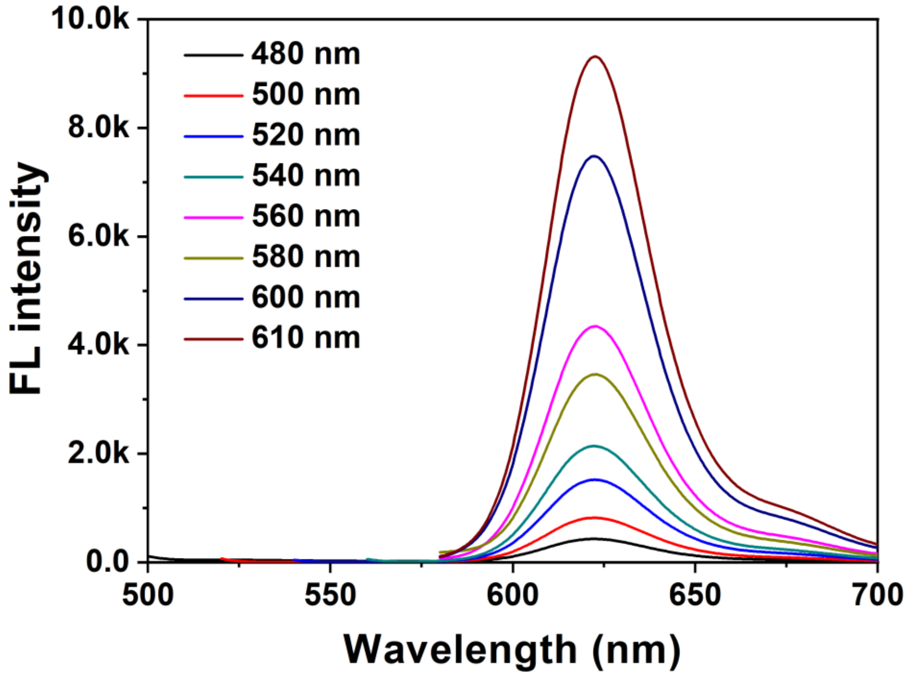

Fig. S 2 | Fluorescence spectra of prepared Red-CDs excited under different wavelength varied from 480 nm to 610 nm.

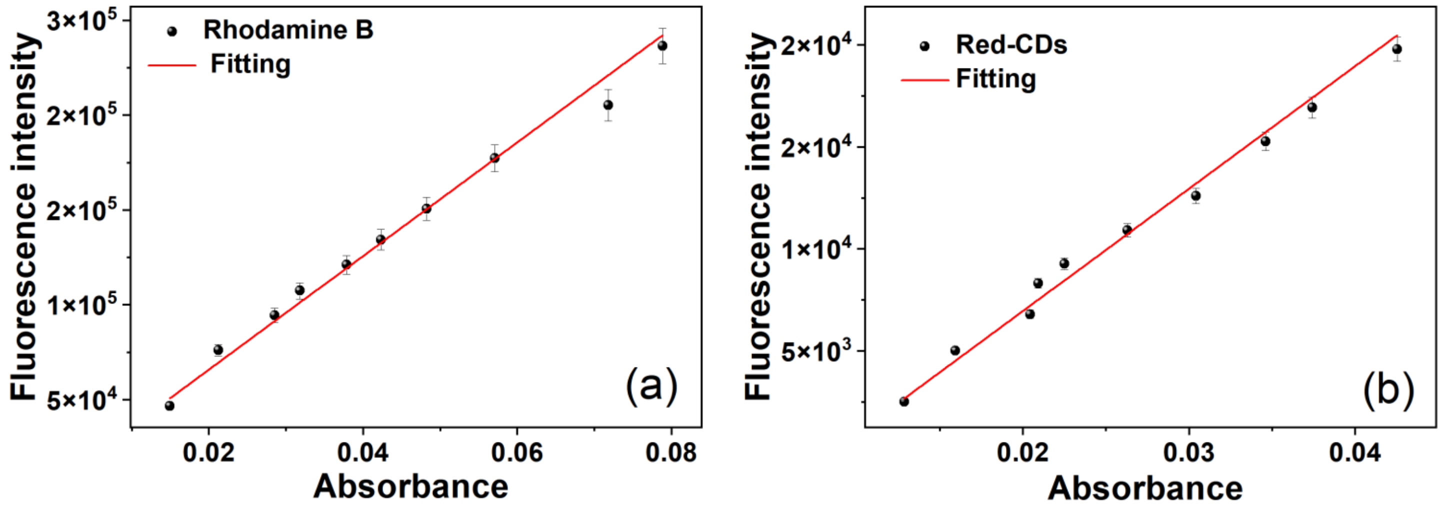

Fig. S 3 | (a) Fluorescence QYs standard curve of Rhodamine B (R^2=0.99); (b) Fluorescence QYs curve of Prepared Red-CDs (R^2=0.99).

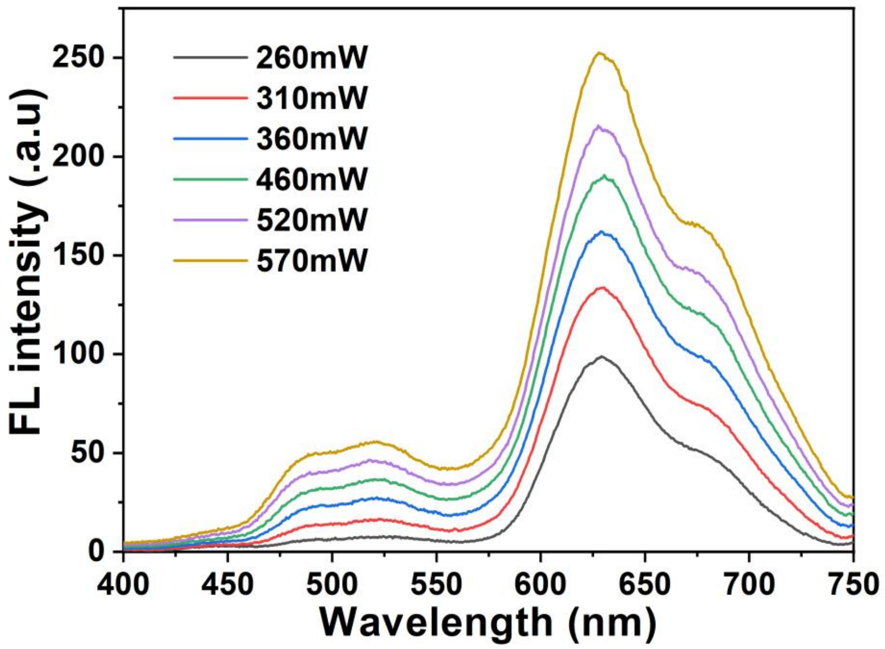

Fig. S 4 | Upconversion fluorescence spectra of the prepared Red-CDs.

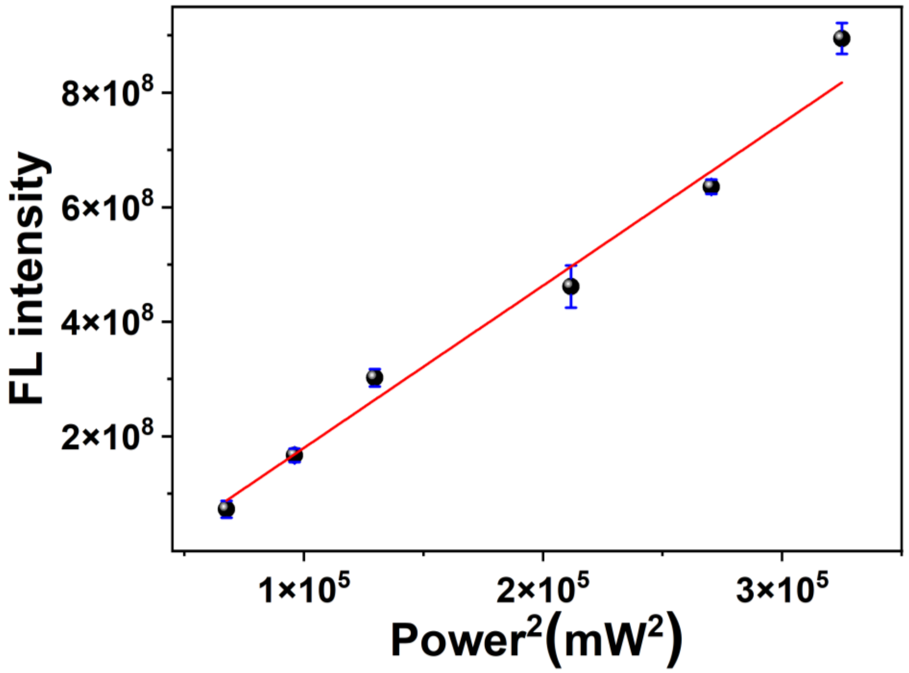

Fig. S 5 | Relationship between upconversion fluorescence intensity and laser power (R^2=0.98).

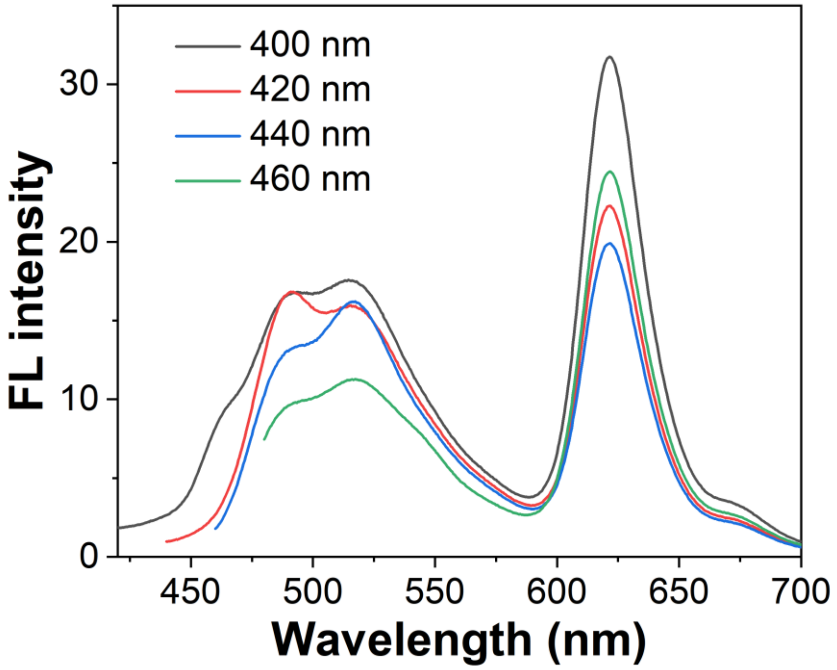

Fig. S 6 | Steady fluorescence spectra of Red-CDs under excitation of 400 nm.

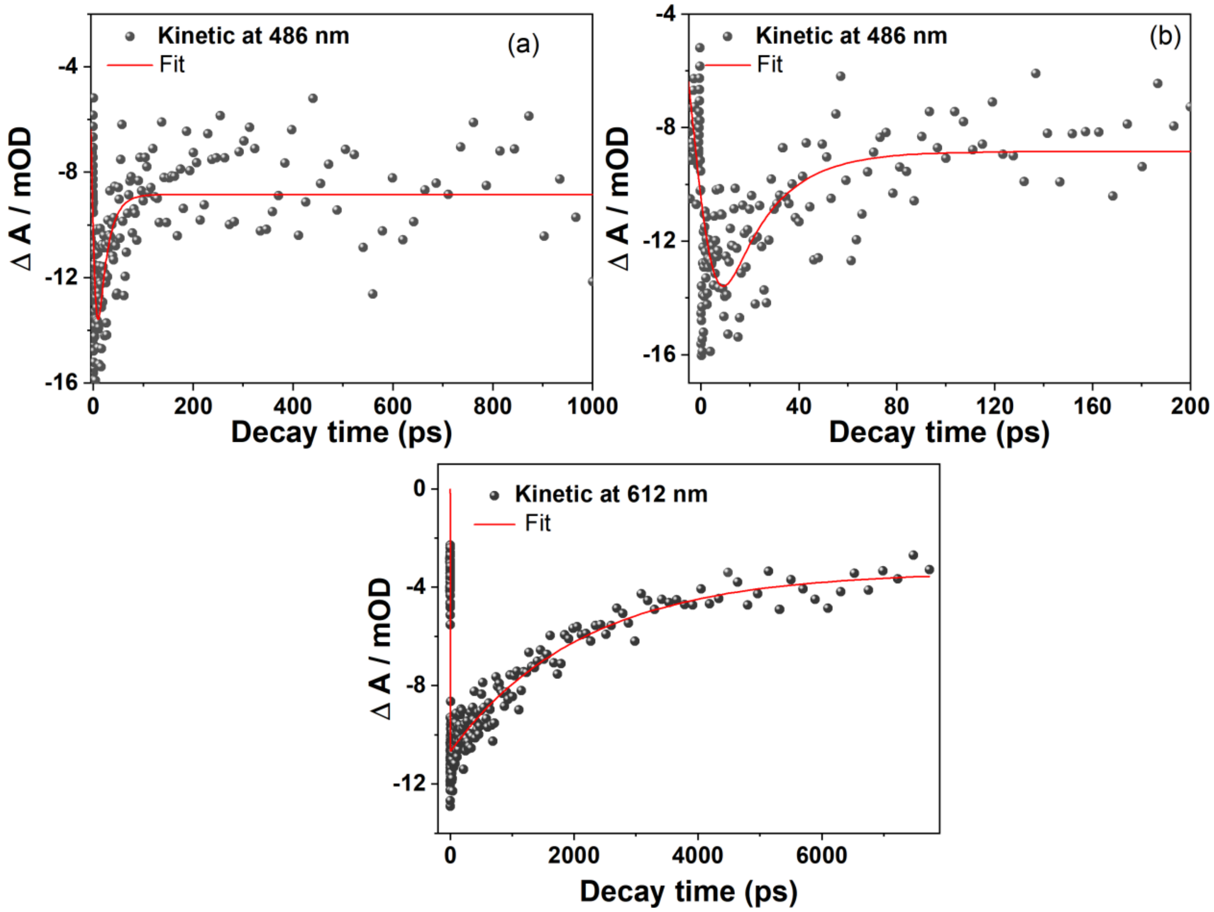

Fig. S 7. kinetic traces of transient absorption spectra at probe wavelength of (a), (b) 486 nm and (c) 612 nm.

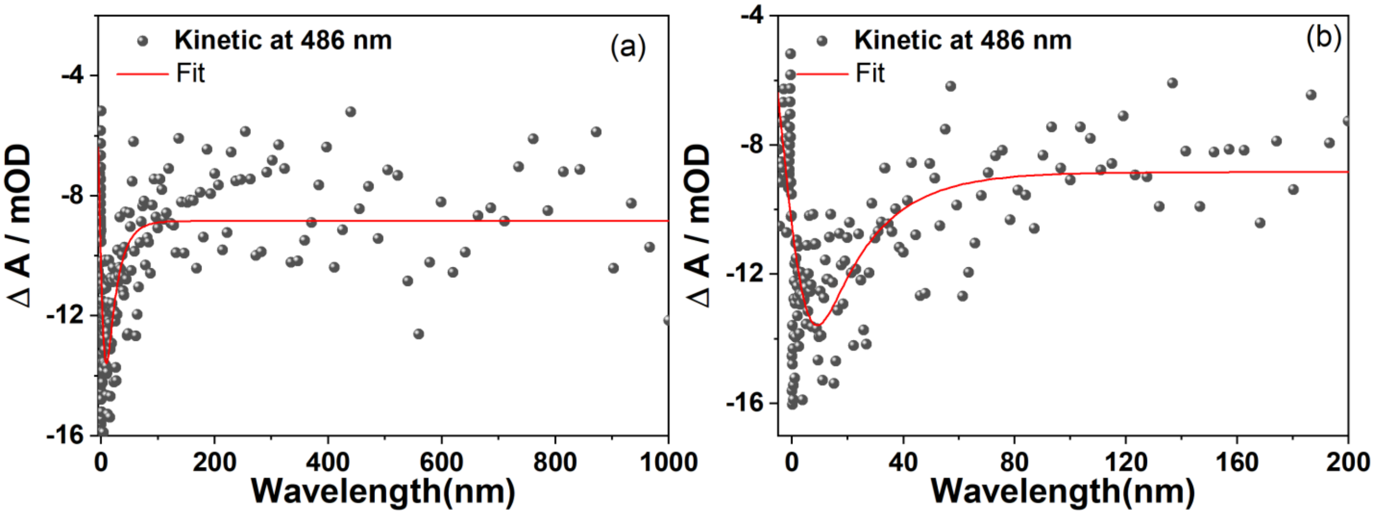

Fig. S 8 | kinetic traces of transient absorption spectra at probe wavelength of 486 nm (curve fitted with standard deviation of 0.00178).

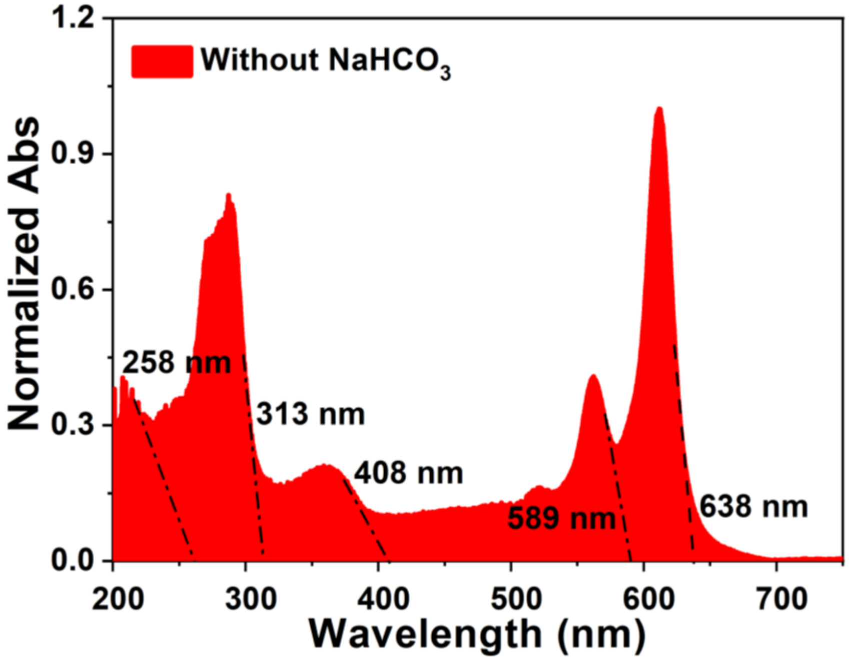

Fig. S 9 | Absorption edge of prepared Red-CDs.

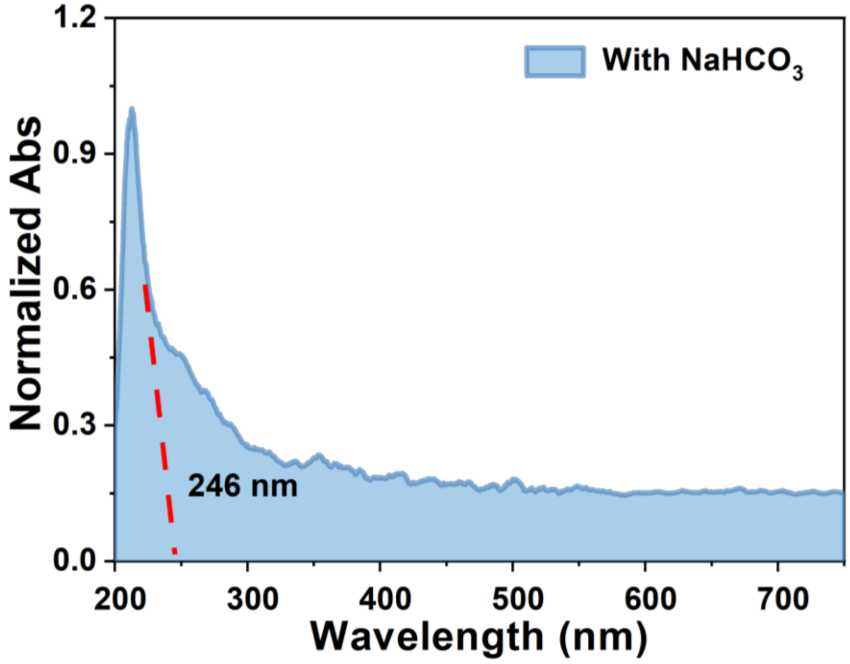

Fig. S 10 | Absorption edge of prepared Red-CDs treated with $NaHCO_3$ solution.

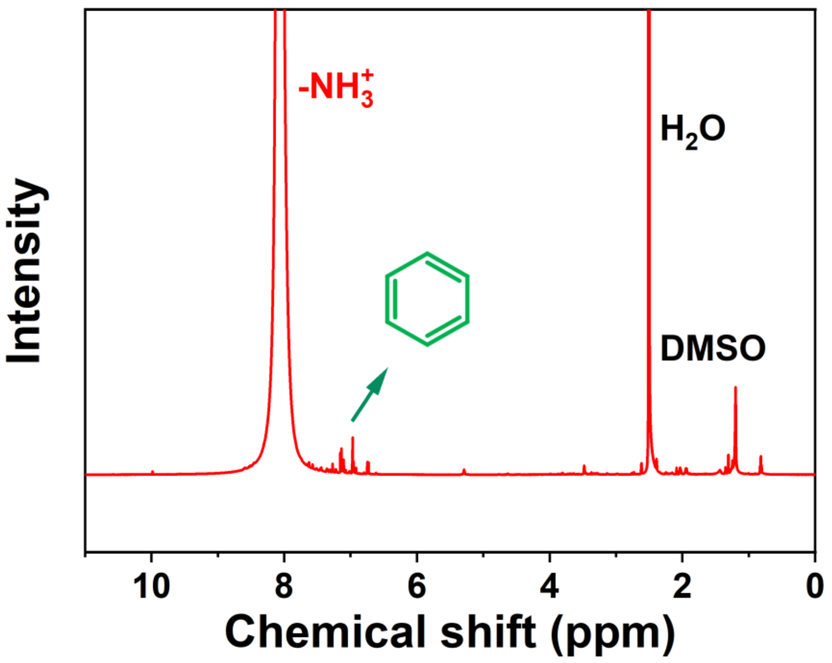

Fig. S 11 | Zoomed H[1]-NMR spectrum of Red-CDs.

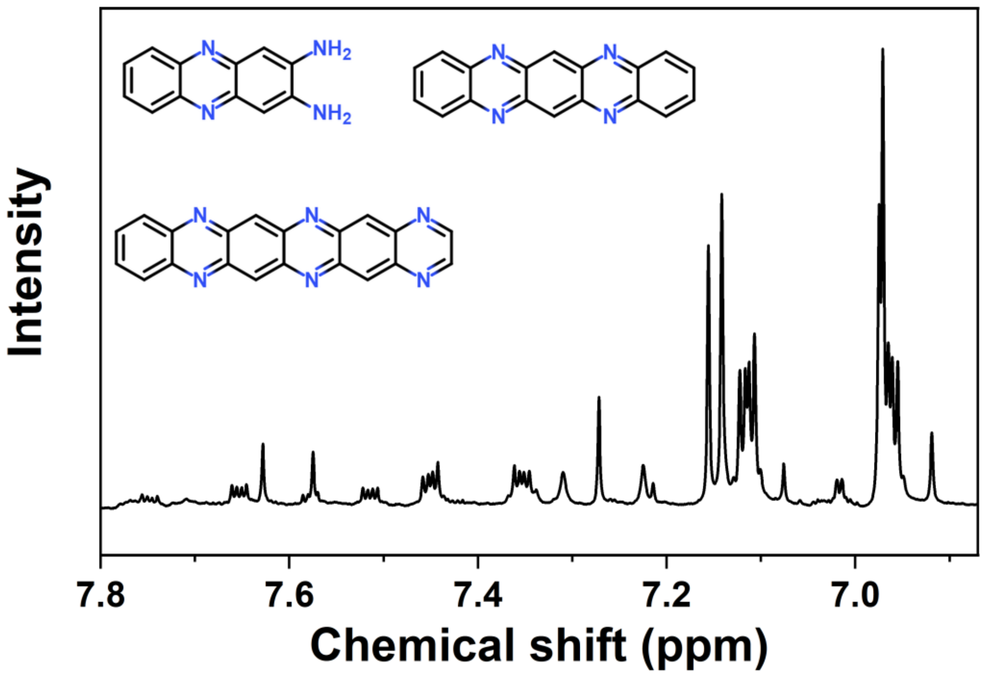

Fig. S 12| Zoomed H[1]-NMR spectrum of Red-CDs between 6.85 ppm to 7.8 ppm.

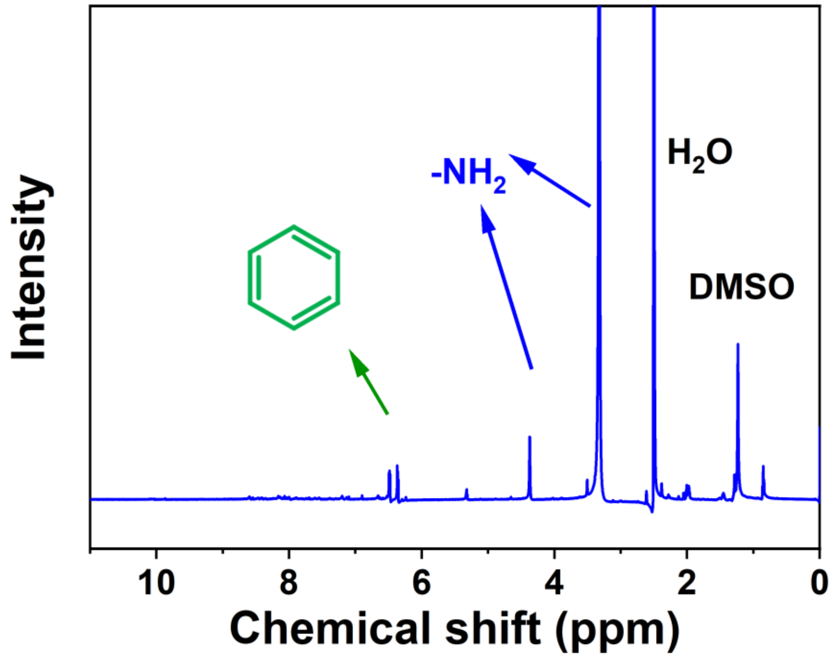

Fig. S 13 | Zoomed H[1]-NMR spectrum of prepared Red-CDs treated with $NaHCO_3$ solution.

			Mean (mV)	Area (%)	St Dev (mV)
Zeta Potential (mV):	38.5	Peak 1:	0.00	0.0	0.00
Zeta Deviation (mV):	0.00	Peak 2:	0.00	0.0	0.00
Conductivity (mS/cm):	348	Peak 3:	0.00	0.0	0.00

Fig. S14 | Zeta potential data of the Red-CDs.

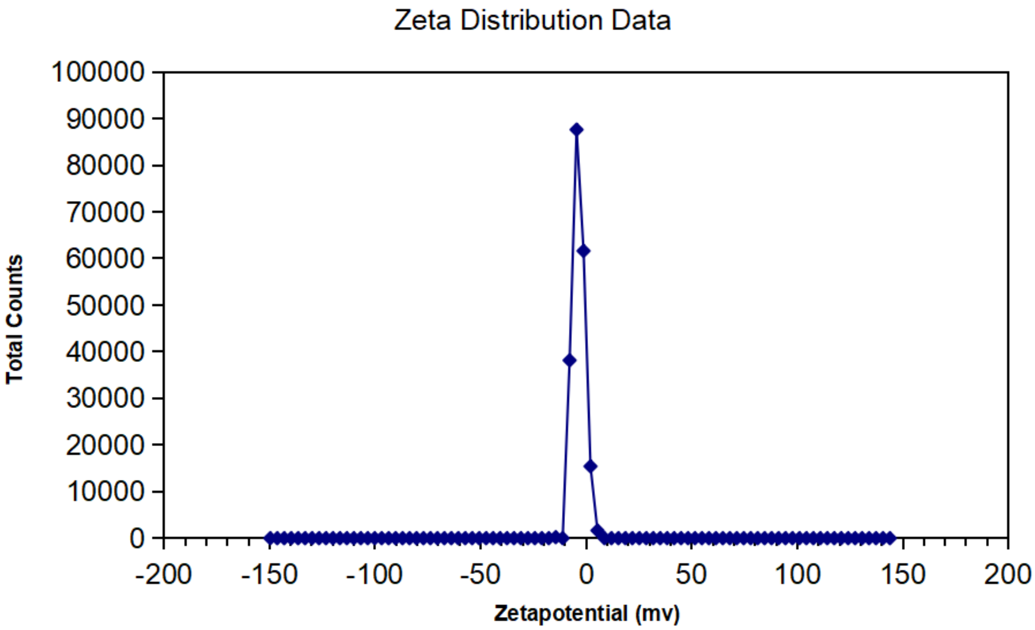

Fig. S 15 | Zeta potential of the Red-CDs treated with $NaHCO_3$

			Mean (mV)	Area (%)	St Dev (mV)
Zeta Potential (mV):	-3.60	Peak 1:	-3.59	99.9	2.91
Zeta Deviation (mV):	2.93	Peak 2:	-14.4	0.1	1.69e-7
Conductivity (mS/cm):	2.96	Peak 3:	0.00	0.0	0.00

Fig. S 16 |Zeta potential data of the Red-CDs treated with $NaHCO_3$

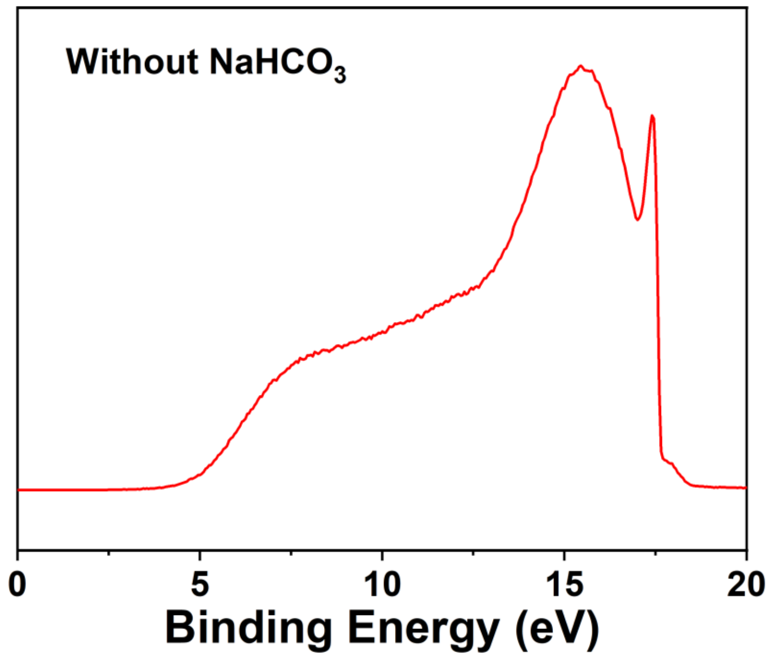

Fig. S 17 | Full scale UPS spectra of prepared Red-CDs.

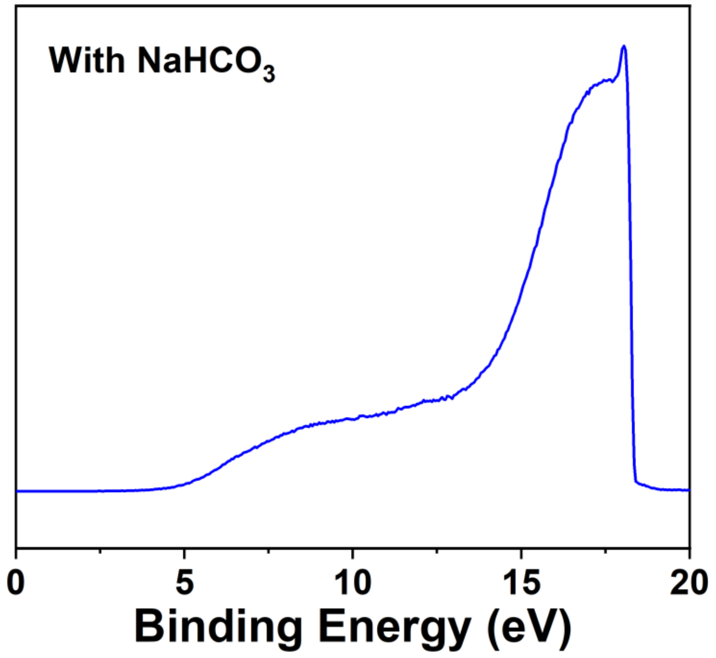

Fig. S 18 | Full scale UPS spectra of prepared Red-CDs treated with $NaHCO_3$ solution.

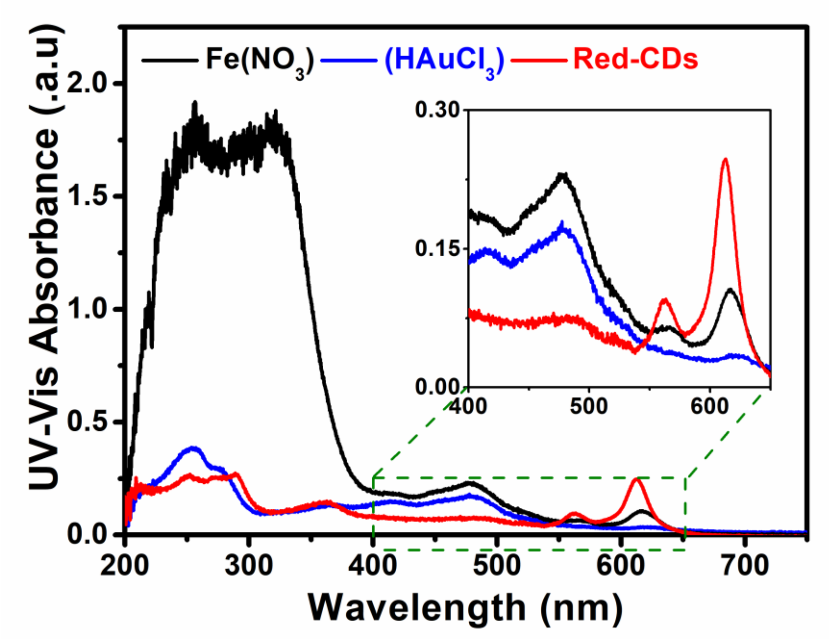

Fig. S 19 | Variations of UV-Vis absorption during the selective detection of Au^{3+} and Fe^{3+} ions.

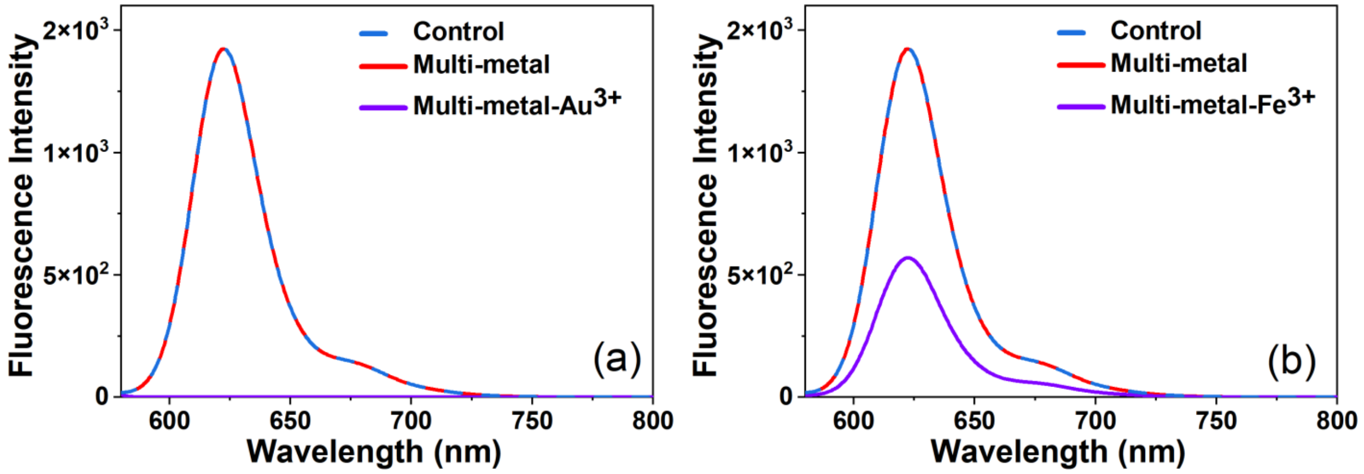

Fig. S 20 | Selective (a) Au^{3+} ions and (b) Fe^{3+} ions detection under inorganic salts interference of $ZnSO_4$, Na_2SO_4, KCl, $MgSO_4$, $NiSO_4$ and $CuSO_4$.

Table S1. Comparison of the FWHM between Red-CDs and the latest literature.

Literatures	Wavelength (nm)	FWHM (nm)
Light: Science & Applications, 2022, 11, 113.[1]	640 nm	75 nm
Nature Biomedical Engineering, 4, 2020, 704. [2]	700 nm	80 nm
Nature Photonics, 2020, 14, 171.[3]	433 nm	35 nm
Journal of Materials Chemistry C, 2019, 7, 3617.[4]	645 nm	106 nm
Nature Communications, 2019, 10, 1789.[5]	615 nm	105 nm
Small, 2018, 14, 1800612.[6]	745 nm	55 nm
Small 2018, 14, 1703919.[7]	630 nm	40 nm
Advanced Materials 2018, 30, 1705913.[8]	640 nm/760 nm	75 nm~120 nm
**This work**	**622 nm**	**33 nm**

Table S2. Comparison of one-photon Red emission of Carbon Dots between previous research and our work.

Reference	Literature	Mechanism of one-photon Red emission	Regulating strategy
1[9]	*Advanced Materials* 2015, 27, 1663	increases of oxidation degree	Not mentioned
2[10]	*Angew.Chem.-Int.Edit.*2015,54, 2970	increases of C-O-C & C-O content	**Reaction condition**
3[11]	*Acs Nano* 2016, 10, 484	increases of COO- content	Not mentioned
4[12]	*Nanoscale* 2016, 8, 729	Increases of N content	**Reactants ratio**
5[13]	*Acs Nano* 2017, 11, 12402.	Increases of graphitic-N content	Not mentioned
6[14]	*Advanced Materials*, 2018, 30.	Increase of graphitization degree and -COOH modification	**Reaction Temperature and reactants ratio**
7[15]	*Advanced Materials*, 2021, 33, 2104872.	Nitrogen-Induced Intramolecular Charge Transfer	**Reactants ratio**
8[16]	*Light: Science & Applications, 2022, 11, 298*	Fluorophore DHQP	Not mentioned
9[17]	*Light: Science & Applications, 2022, 11, 172*	Electron–phonon coupling	Not mentioned
**Our work**		**Protonation of -NH_2 conjugated with pyridinic structure**	**Protonation/Deprotonation**

(Note: 5,14-dihydroquinoxalino[2,3-b] phenazine, DHQP**)**

Table S3 Comparison of two-photon Red emission mechanism of CDs between previous research and our work.

Reference	Literature	Mechanism of two-photon Red emission	Regulating strategy
1[18]	*J. Mater. Chem.* 2012, 22	Electron-transition promoted by nitrogen doping (Speculation without evidence).	Not mentioned
2[19]	*Nano Lett. 2013,* 13, 2436.	Electron-transition between $-NH_2$ and π- conjugated systems of GQDs. (Speculation without evidence)	Not mentioned
4[20]	*Carbon* 2015, 81, 367.	Excitation cross section of defect states or size distribution.	Not mentioned
5[21]	*Advanced Materials* 2017, 29	Electron-transition of Donor (D) - π - (A) acceptor system consisting of surface functional groups and carbon framework of CDs.	Not mentioned
3[22]	*ACS Sustain. Chem. Eng.* 2018, 6, 4711.	Electron-transition between OH, $-NH_2$ groups and π-conjugated systems of GQDs. (Speculation without evidence)	Not mentioned
6[8]	*Advanced Materials* 2018, 30.	Electron-transitions promoted by the enhanced electron acceptor C=O/S=O groups modified on CDs surface.	Not mentioned
8[23]	*Small 2020, 2000680*	Nitrogen adoption	**Nitrogen adoption**
8[24]	*Advanced Science, 2022, 9, 2202283*	Precursors molecular conjugation	**Bovine serum albumin adsorption**
9[25]	*Nano Research. 2022, 15, 9470.*	Graphitic-N or amino-N	Not mentioned
**Our work**		**Protonation of $-NH_2$ conjugated with pyridinic structure**	**Protonation/Deprotonation**

Table S4. Fluorescence QYs data of Rhodamine B and Red-CDs.

Materials	Slope	QYs
Rhodamine B	2995523.64	85%
Red-CDs	600335.5438	17%

Reference

[1] H. Zhang, G. Wang, Z. Zhang, J. H. Lei, T.-M. Liu, G. Xing, C.-X. Deng, Z. Tang, S. Qu, *Light: Science & Applications* **2022**, 11, 113.

[2] S. H. Li, W. Su, H. Wu, T. Yuan, C. Yuan, J. Liu, G. Deng, X. C. Gao, Z. M. Chen, Y. M. Bao, F. L. Yuan, S. X. Zhou, H. W. Tan, Y. C. Li, X. H. Li, L. Z. Fan, J. Zhu, A. T. Chen, F. Y. Liu, Y. Zhou, M. Li, X. C. Zhai, J. B. Zhou, *Nature Biomedical Engineering* **2020**, 4, 704.

[3] F. L. Yuan, Y. K. Wang, G. Sharma, Y. T. Dong, X. P. Zheng, P. C. Li, A. Johnston, G. Bappi, J. Z. Fan, H. Kung, B. Chen, M. I. Saidaminov, K. Singh, O. Voznyy, O. M. Bakr, Z. H. Lu, E. H. Sargent, *Nature Photonics* **2020**, 14, 171.

[4] M. C. Li, X. J. Zhang, H. R. Zhang, W. B. Chen, L. Ma, X. J. Wang, Y. L. Liu, B. F. Lei, *J. Mater. Chem. C* **2019**, 7, 3617.

[5] H. Yang, Y. Liu, Z. Guo, B. Lei, J. Zhuang, X. Zhang, Z. Liu, C. Hu, *Nature Communications* **2019**, 10, 1789.

[6] H. Ding, J. S. Wei, P. Zhang, Z. Y. Zhou, Q. Y. Gao, H. M. Xiong, *Small* **2018**, 14, 10, 1800612.

[7] J. J. Liu, D. W. Li, K. Zhang, M. X. Yang, H. C. Sun, B. Yang, *Small* **2018**, 14, 10, 1703919.

[8] D. Li, P. T. Jing, L. H. Sun, Y. An, X. Y. Shan, X. H. Lu, D. Zhou, D. Han, D. Z. Shen, Y. C. Zhai, S. N. Qu, R. Zboril, A. L. Rogach, *Advanced Materials* **2018**, 30, 1705913.

[9] L. Bao, C. Liu, Z. L. Zhang, D. W. Pang, *Advanced Materials* **2015**, 27, 1663.

[10] S. L. Hu, A. Trinchi, P. Atkin, I. Cole, *Angew. Chem.-Int. Edit.* **2015**, 54, 2970.

[11] H. Ding, S.-B. Yu, J.-S. Wei, H.-M. Xiong, *Acs Nano* **2016**, 10, 484.

[12] L. Guo, J. Ge, W. Liu, G. Niu, Q. Jia, H. Wang, P. Wang, *Nanoscale* **2015**, 8, 729.

[13] K. Hola, M. Sudolska, S. Kalytchuk, D. Nachtigallova, A. L. Rogach, M. Otyepka, R. Zboril, *Acs Nano* **2017**, 11, 12402.

[14] X. Miao, D. Qu, D. X. Yang, B. Nie, Y. K. Zhao, H. Y. Fan, Z. C. Sun, *Advanced Materials* **2018**, 30, 1704740.

[15] X. Xu, L. Mo, Y. Li, X. Pan, G. Hu, B. Lei, X. Zhang, M. Zheng, J. Zhuang, Y. Liu, C. Hu, *Advanced Materials* **2021**, 33, 2104872.

[16] P. F. Li, S. S. Xue, L. Sun, X. P. Zong, L. An, D. Qu, X. Y. Wang, Z. C. Sun, *Light-Sci. Appl.* **2022**, 11, 11, 298.

[17] B. Wang, Z. Wei, L. Sui, J. Yu, B. Zhang, X. Wang, S. Feng, H. Song, X. Yong, Y. Tian, B. Yang, S. Lu, *Light: Science & Applications* **2022**, 11, 172.

[18] C. F. Wang, X. Wu, X. P. Li, W. T. Wang, L. Z. Wang, M. Gu, Q. Li, *Journal of Materials Chemistry* **2012**, 22, 15522.

[19] Q. Liu, B. Guo, Z. Rao, B. Zhang, J. R. Gong, *Nano Letters* **2013**, 13, 2436.

[20] H. D. Ha, M.-H. Jang, F. Liu, Y.-H. Cho, T. S. Seo, *Carbon* **2015**, 81, 367.

[21] S. Y. Lu, L. Z. Sui, J. J. Liu, S. J. Zhu, A. M. Chen, M. X. Jin, B. Yang, *Advanced Materials* **2017**, 29, 1603443.

[22] L. Wang, W. T. Li, M. Li, Q. Q. Su, Z. Li, D. Y. Pan, M. H. Wu, *Acs Sustainable Chemistry & Engineering* **2018**, 6, 4711.

[23] L. Jiang, H. H. Ding, M. S. Xu, X. L. Hu, S. L. Li, M. Z. Zhang, Q. Zhang, Q. Y. Wang, S. Y. Lu, Y. P. Tian, H. Bi, *Small* **2020**, 16, 9, 2000680.
[24] Y. P. Liu, J. H. Lei, G. Wang, Z. M. Zhang, J. Wu, B. H. Zhang, H. Q. Zhang, E. S. Liu, L. M. Wang, T. M. Liu, G. C. Xing, D. F. Ouyang, C. X. Deng, Z. K. Tang, S. N. Qu, *Adv. Sci.* **2022**, 9, 11, 2202283.
[25] X. Y. Zhou, K. B. Yi, Y. L. Yang, G. H. Xie, X. H. Ji, Z. K. He, *Nano Res.* **2022**, 15, 9470.
